# Proteomic Characterization of *Bradyrhizobium diazoefficiens* Bacteroids Reveals a Post-Symbiotic, Hemibiotrophic-Like Lifestyle of the Bacteria within Senescing Soybean Nodules

**DOI:** 10.3390/ijms19123947

**Published:** 2018-12-08

**Authors:** Kent N. Strodtman, Sooyoung Frank, Severin Stevenson, Jay J. Thelen, David W. Emerich

**Affiliations:** 1 Department of Science, Columbia College, Columbia, MO 65216, USA; knstrodtman@ccis.edu; 2 Department of Biochemistry, University of Missouri, Columbia, MO 65211, USA; sooyoungfranck@live.com (S.F.); ThelenJ@Missouri.edu (J.J.T.); 3 Charles River Labs, Carson City, NV 89523, USA; severin.stevenson@gmail.com

**Keywords:** *Bradyrhizobium diazoefficiens*, soybean, *Glycine max*, nitrogen fixation, senescence, proteomics, hemibiotroph

## Abstract

The form and physiology of *Bradyrhizobium diazoefficiens* after the decline of symbiotic nitrogen fixation has been characterized. Proteomic analyses showed that post-symbiotic *B. diazoefficiens* underwent metabolic remodeling as well-defined groups of proteins declined, increased or remained unchanged from 56 to 119 days after planting, suggesting a transition to a hemibiotrophic-like lifestyle. Enzymatic analysis showed distinct patterns in both the cytoplasm and the periplasm. Similar to the bacteroid, the post-symbiotic bacteria rely on a non-citric acid cycle supply of succinate and, although viable, they did not demonstrate the ability to grow within the senescent nodule.

## 1. Introduction

In 1879, the German mycologist Heinrich Anton de Bary, defined symbiosis as “the living together of unlike organisms” [1]. Nitrogen-fixing symbioses between rhizobia and legumes have been studied since 1888 [2], with the vast number of investigations describing the infection events and the mature nitrogen-fixing nodule. During nodule formation, the rhizobia transform into a non-growing form capable of reducing atmospheric dinitrogen, called bacteroids. The plant receives reduced nitrogen compounds in exchange for photosynthetically-derived substrates transported to the bacteroids to provide the energy for the nitrogen-fixing reactions.

In determinate nodules, such as those formed between *Bradyrhizobium diazoefficiens* and soybean, the nitrogen fixation activity of the nodule increases in parallel with nodule development and then declines as the plant portion of the nodule senesces. With bacteroids obtained from senescing, determinate nodules are able to de-differentiate into free-living bacteria and thus remain viable [3,4,5,6,7]. Bacteroids within the decaying nodule could take advantage of the abundant supply of metabolites from the decaying plant nodule, in effect becoming hemibiotrophs. A hemibiotroph is an organism that is a saprophyte or parasite in living tissue while the plant is alive, and which upon plant death consumes the decaying tissue [8,9]. According to the original definition of Anton de Bary [1], the senescing nodule is no longer a symbiosis, since the unlike organisms are no longer living together, but rather one is surviving on the remains of the other. This post-symbiotic, hemibiotrophic-like lifestyle of the bradyrhizobia has received scant attention, but has significant ecological relevance, as it may be the primary mechanism by which the bacteria are perpetuated in the rhizosphere and soil. The rhizosphere supports a far greater number of bacteria than the bulk soil [10] because up to 20% of the entire carbon fixed photosynthetically by the plant may be excreted from the roots [11].

Unlike the symbiotic state, in which the symbiotic bacteroids are provide a defined diet of substrates dictated by the plant, the post-symbiotic bacteria are presented with a diverse milieu of metabolites derived from the catabolism of the entire cellular content of plant nodule cells. In contrast to the rhizosphere, where bacteria must compete for excreted materials, the bradyrhiobia are imbedded within a rich metabolic matrix, for which they do not need to compete. Elucidating the genes and molecular events for survival and perpetuation of applied strains beyond symbiosis in the senescent nodule and their eventual release into the soil would be an agricultural and financial benefit to farmers in third world-countries, who lack the resources for annual fertilizer applications.

Proteomic and transcriptomic analysis of *Bradyrhizobium diazoefficiens* bacteroids has been undertaken to better understand the symbiosis between *B. diazoefficiens* and its obligate legume host soybean (*Glycine max*) to improve crop production [12,13]. However, the majority of this work has only focused on the early stages of infection to the peak of symbiotic nitrogen fixation. Though much is known about the process of nodule senescence with regard to the plant, little is known about the determinate bacteroid and its process of post-symbiotic re-differentiation [6,13,14,15,16]. Only one published proteomics report examines bacteroids past the peak of nitrogen fixation and utilizing soybean root nodules grown under field conditions [12]. This leaves a glaring omission in the critical stage in the natural cycle where the bradyrhizobia return to the soil. This study was undertaken to provide a global proteomic analysis of the post-symbiotic form of *B. diazoefficiens.* Purified bacteroids were fractionated into their periplasmic and cytoplasmic compartments and marker enzymes were followed over a period of 9 weeks. The fractionated proteins were prepared for analysis via LC-MS/MS and three general patterns were identified: Proteins decreasing in abundance, constitutive proteins, and proteins increasing in abundance. The results of this study should help in understanding how the *B. diazoefficiens* persists after symbiosis to provide greater insight into how the association could be better exploited to increase crop production.

## 2. Results

### 2.1. Nodule Mass and Leghemoglobin Content

**Figure 1:**
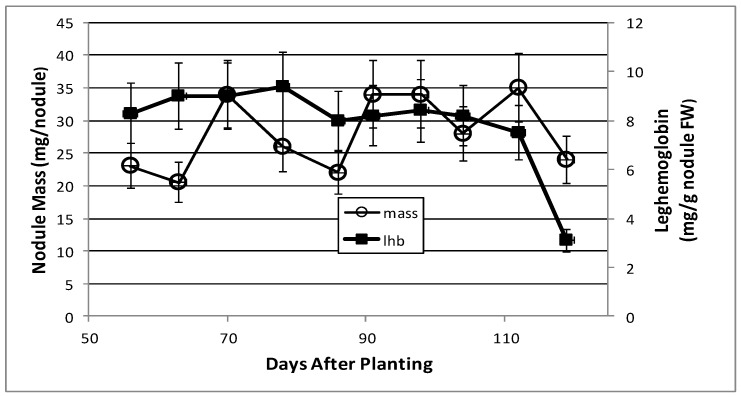
Soybean nodule mass and leghemoglobin content from soybean nodules at various days after planting. The values are the mean ± standard deviation of three replicates.

Soybean root nodules were measured for mass per nodule and leghemoglobin content over the 9-week (56–119 days after planting) post-symbiotic period. The maximal nitrogen fixation activity was observed on day 43, but by day 55 it had declined to 25% and was negligible by day 95 (data not shown). Nodule mass fluctuated over time, but the leghemoglobin content was consistently between 8–9 mg of leghemoglobin per g fresh weight of nodules until day 112, when leghemoglobin concentration started to decline, with a final concentration of 3 mg per g nodule by day 119 (Figure 1).

### 2.2. Bacteroid Protein and Poly-β-hydroxybutyrate (PHB) Content and Enzymes Activities in the Post-Symbiotic Period

**Figure 2:**
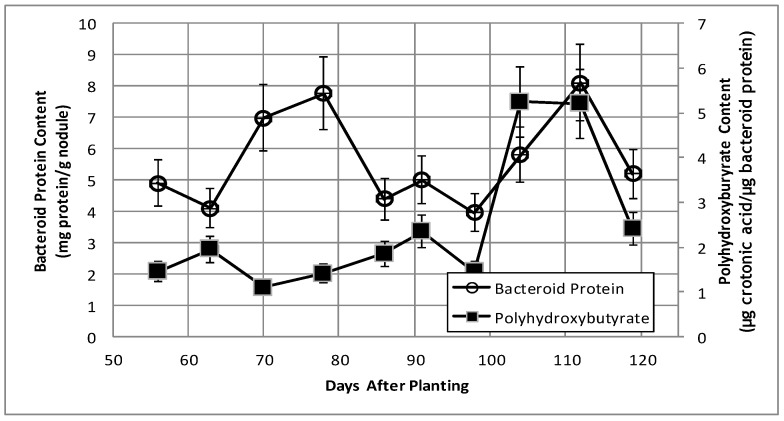
Bacteroid protein content and polyhydroxybutyate content of bacteroids isolated from soybean nodules at various days after planting. The values are the mean ± standard deviation of three replicates.

**Figure 3:**
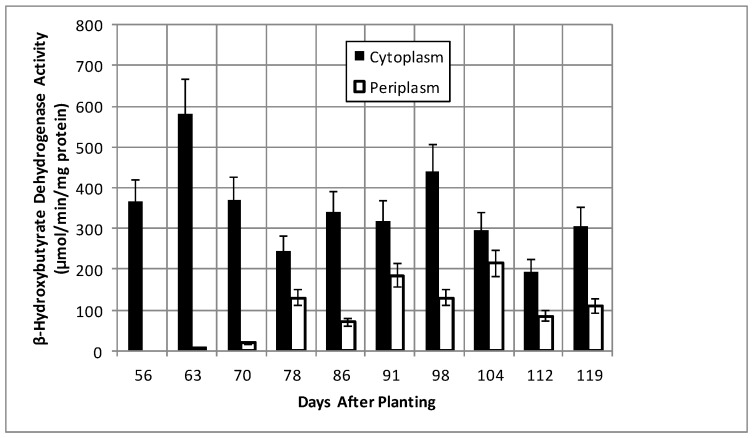
β-Hydroxybutyrate dehydrogenase activity of bacteroids isolated from soybean nodules at various days after planting. The values are the mean ± standard deviation of three replicates.

**Figure 4:**
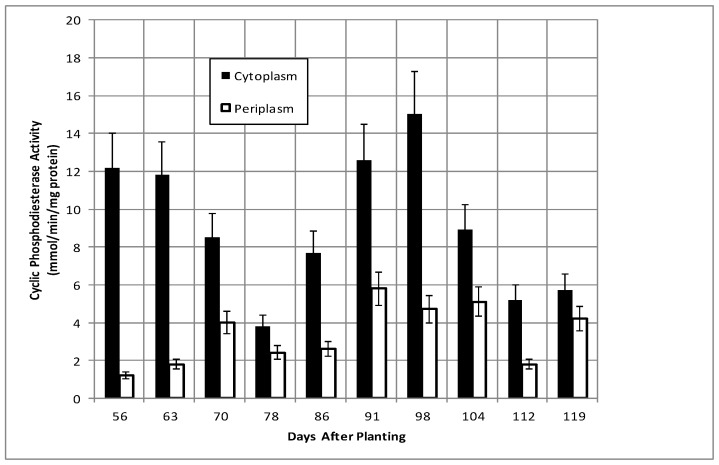
Cyclic phosphodiesterase activity of bacteroids isolated from soybean nodules at various days after planting. The values are the mean ± standard deviation of three replicates.

Total bacteroid protein fluctuated over the time course with a pattern similar to, but not identical with, that of nodule mass (Figure 2). Isolated bacteroids were fractionated into periplasmic and cytoplasmic fractions. The periplasm is at the interface between the bacteria and the plant and, thus, would be assumed to respond to changes caused by the post-symbiotic environment. β-hydroxybutyrate dehydrogenase, a cytoplasmic enzyme marker necessary for the production of polyhydroxybutyrate (PHB), a bacteroid carbon storage polymer associated with effective symbiosis, displayed cytoplasmic activity, remaining relatively constant, and periplasmic activity increased to 91 days and remained relatively constant until it declined at days 112 and 119 (Figure 3). The PHB content remained relatively unchanged until days 104–112, when it increased nearly 3-fold (Figure 2). The periplasmic marker enzyme cyclic phosphodiesterase displayed a bimodal pattern, while the periplasmic activity increased from day 55 to 91 and then remained constant (Figure 4).

**Figure 5:**
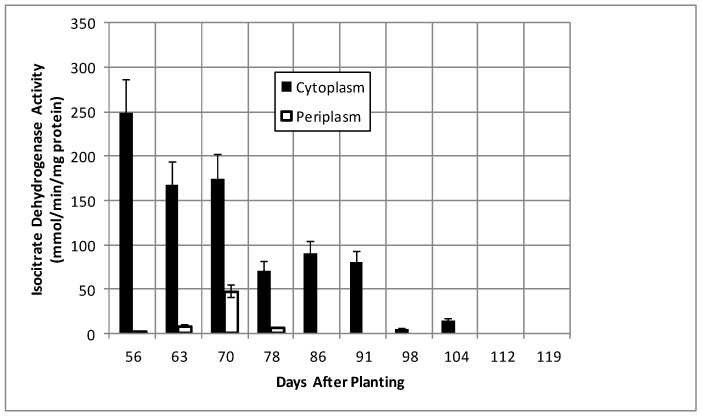
Isocitrate dehydrogenase activity of bacteroids isolated from soybean nodules at various days after planting. The values are the mean ± standard deviation of three replicates.

**Figure 6:**
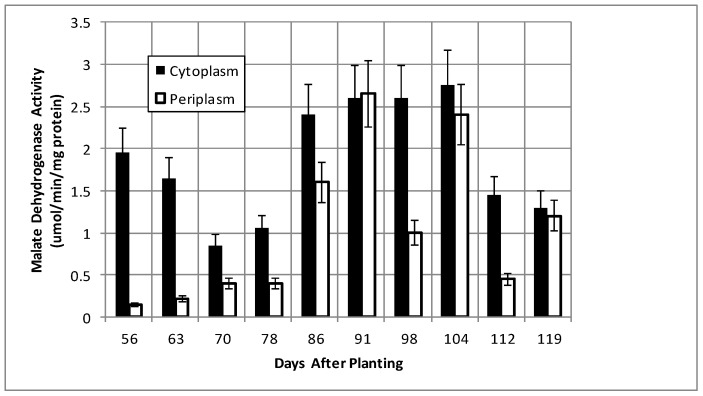
Malate dehydrogenase activity of bacteroids isolated from soybean nodules at various days after planting. The values are the mean ± standard deviation of three replicates.

**Figure 7:**
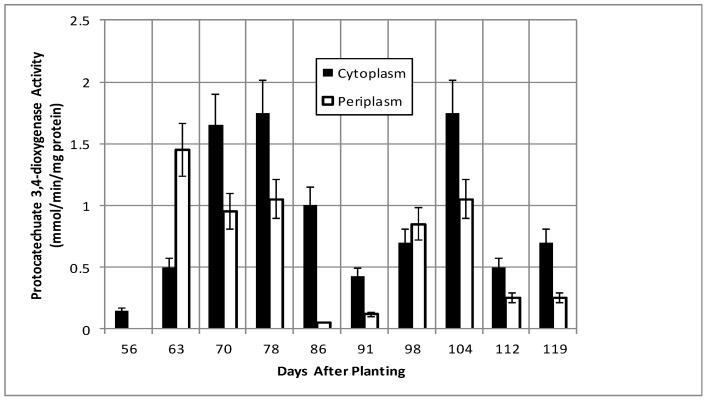
Protocatechuate 3,4-dioxygenase activity of bacteroids isolated from soybean nodules at various days after planting. The values are the mean ± standard deviation of three replicates.

Isocitrate dehydrogenase, another cytoplasmic marker enzyme, has been previously shown to decline over the first five weeks of symbiosis [17,18] and Figure 5 shows it continued to decline and became undetectable at days 112 and 119. Cytoplasmic malate dehydrogenase activity showed a bimodal trend similar to cyclic phosphodiesterase activity and the periplasmic malate dehydrogenase activity showed a gradual increase through 78 days and then a more pronounced increase to 91 days and a decrease at days 112 and 119 (Figure 6). Protocatechuate 3,4-dioxygenase activity in both fractions showed a bimodal activity profile (Figure 7).

### 2.3. Proteomics Time Course

**Figure 8:**
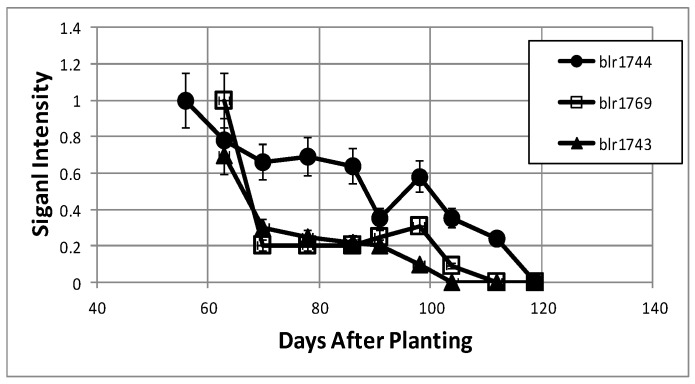
Examples of bacteroid proteins displaying declining signal intensities as a function of days after planting. The values are the mean ± standard deviation of twelve values (six biological and two technical replicates).

**Figure 9:**
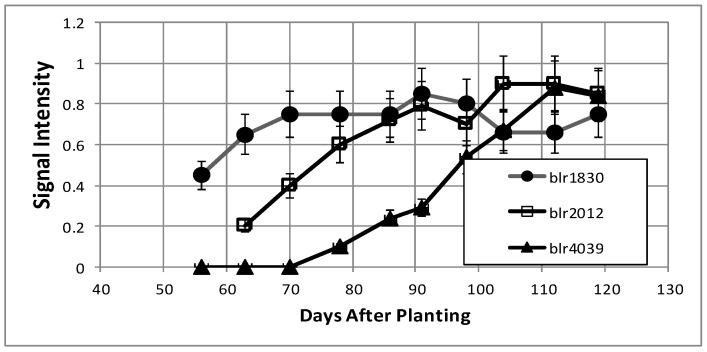
Examples of bacteroid proteins displaying increasing signal intensities as a function of days after planting. The values are the mean ± standard deviation of twelve values (six biological and two technical replicates).

LC-MS/MS analysis was performed on the proteins of the cytosolic and periplasmic fractions of bacteroids isolated from soybean plants over the nine-week time course. Periplasmic protein samples covered the entirety of the time course, while cytoplasmic analysis covered the seven time points of days 63, 70, 91–119. For the cytosolic fraction, 1869 unique peptides were identified, with 706 proteins identified via SePro. For the periplasmic fraction, 2849 peptides were identified, with 1417 proteins identified via SePro. Trend Quest from Pattern Lab for Proteomics identified three unambiguous progressions of peptide frequencies: Proteins that declined following symbiosis, proteins that increased following symbiosis, and constitutive proteins (Figure 8 and Figure 9). Other patterns displayed significant fluctuations at various sampling times that are difficult to interpret. These proteins may be more responsive to climatic or soil conditions than those of the three unambiguous patterns. The sampling time points include the development of nitrogen fixation activity and proteins known to be involved in this process were identified. Proteins known to participate in nodule initiation were absent and likely have been degraded as they have served their purpose at the first sampling point of functional nodules, actively reducing atmospheric dinitrogen. Proteins associated with symbiotic nitrogen fixation were identified: The nitrogenase metallo cluster biosynthetic protein (blr1756), nitrogenase molybdenum-cofactor synthesis protein (blr1746), nitrogenase stabilizing protein (blr1771), glutathione synthetase (bll0668), alanine dehydrogenase (blr3179) alanine racemase (bll4070), serine hydroxymethyltransferase (bll5033), L-asparaginase (bll4950), aspartate-semialdehyde dehydrogenase (bll0501), and aspartate aminotransferase (bll7416). All of these proteins declined markedly during senescence.

### 2.4. Proteins That Declined Following Symbiosis

The rate of protein synthesis and protein turnover have been shown to decline during nodule development due to the diversion of cellular energy to nitrogen fixation [19] and, as expected in the post-symbiotic period, proteins directly associated with nitrogen fixation, the two component proteins of nitrogenase (blr1743, blr1744), and fixC, a flavoprotein dehydrogenase (blr1774), were found to decrease over the nine-week time course (Table 1). All three proteins are regulated by RegR under microoxic conditions [20,21]. The ability to assimilate fixed nitrogen into transferable amino acids decreased over time as the aminotransferase proteins (blr1686, blr4134), glutamate synthase (blr7743), glutamine synthetase I (blr4949), and two enzymes for branched chain amino acid production, 3-isopropylmalate dehydrogenase (bll0504), and 3-isopropylmalate isomerase (blr0488) all decreased over the time course. Succinate semi-aldehyde dehydrogenase (blr0807), which is necessary for the breakdown of glutamate and phenylalanine to succinate [22], also declined.

Proteins of glycolysis and gluconeogensis were well represented in the decreasing data set; pyruvate dehydrogenase (bll4782), phosphoenolpyruvate carboxykinase (bll8141), fructose bisphosphate aldolase (bll1520), and enolase (bll4794). Pyruvate dehydrogenase (bll4782) provides a link between glycolysis to branched chain amino acid biosynthesis. Citric acid cycle enzymes succinyl-CoA synthetase (bll0455) and succinate dehydrogenase (blr0514) were found to decrease over time as well, indicating the decreases in cellular energy needs for nitrogen fixation and the need for carbon backbones for the production of amino acids.

A large number of proteins associated with the ribosome were found to decline. The symbiotic specific GroEL/S3 (blr2059, blr2060) were notable as they serve as a marker of the decline of the symbiotic state of the bacteroid, as GroEL/S3 were induced during the symbiotic state and are regulated by *NifA* [23,24]. The decline of several proteases, LA protease (bll4942), serine transmembrane protease (bll6508), and a zinc protease (blr7485), may suggest a physiological adaptation following symbiosis. The 30S ribosomal proteins S1, S4, S7, and S18 and the 50S ribosomal proteins L14 all decreased beyond 91 days.

### 2.5. Proteins That Increased Following Symbiosis

The number of proteins found to be increasing over the time course (Table 2) was much lower than that for the proteins in decline (Table 1). Half of the proteins associated with this pattern were unknown or hypothetical proteins. Annotated proteins in this pattern include fatty acid metabolism proteins enoyl-CoA hydratase (blr1160), acetyl-CoA carboxylase (blr0191), acyl-CoA thiolase (blr1159), and enoyl-CoA hydratase (bll7821). CheY (bll7795), a two-component transcriptional regulator which was found to be expressed during times of desiccation stress [25], increased over the time course, as did a carboxy-terminal protease (blr0434) and a peptidyl cis-trans isomerase (bll4690), which is required for proper protein folding. Among the proteins without annotation, bll2012 and blr1830 were found to be induced by soybean seed extracts [26].

### 2.6. Constitutive Proteins

The proteins in this group include a set involved in nitrogen metabolism: An ABC transporter substrate binding protein for oligopeptides (bll2909, bll5596, bll7921), a peptidyl-dipeptidase (bll7756), a glycine hydroxymethyltransferase (bll5033), a probable amino acid binding protein (bll2909), a glu/leu/phe/val dehydrogenase (blr2146), which causes the reversible oxidative deamination of the substrate to its respective α-keto acid, an ATP dependent protease, ClpB (blr1404), and N-utilization substance protein (bll0785) (Table 3). A glycine hydroxymethyltransferase, involved in C1 metabolism, can be coupled to the degradation of vanillate, which would be further metabolized via the β-ketoadipate pathway with final cleavage to acetyl-CoA and succinyl-CoA via β-ketoadipyl CoA thiolase (blr0925), which is known to be constitutively expressed in *B. diazoefficiens* [27]. In addition to β-ketoadipyl CoA thiolase, the proteins involved in carbon metabolism include the pentose phosphate enzymes transketolase (bll1524) and transaldolase (blr6758), which provides a range of metabolites and a mechanism for redox cofactor balancing [28,29,30,31,32], methymalonate-semialdehyde dehydrogenase (blr3954), which participates in active turnover in branched chain amino acids and propanoate metabolism, leading to a source of acetyl-CoA, and propanoyl-CoA, a putative alcohol dehydrogenase precursor (bll6220) and carbonic anhydrase (bll2065). Superoxide dismutase (bll7774) and alkyl hydroperoxide reductase (bll1777), proteins involved in reactive oxygen metabolism, were found. Several soybean proteins, histone H4, histone H3, and glu/leu/phe/val dehydrogenase (Glyma02g38920.1, Glyma06g32880.1, Glyma16g04560.1), were found throughout the period of study.

## 3. Discussion

Bacteroid is the term that refers to the symbiotic, nitrogen-fixing form of rhizobia. Franck et al. have demonstrated that post-symbiotic bacteroids are transcriptionally active up to 95 days after planting [33]. The data collected over the period of time from 56 to 119 days after planting clearly demonstrate the metabolic activity of the bacteria that reside within the decaying plant nodule. The bacteria, although possessing enzyme activity, do not possess nitrogenase activity, the central metabolic activity of the symbiosis and, furthermore, the symbiosis no longer occurs as per the definition of Anton de Bary [1], who defined symbiosis as “the living together of unlike organisms”. Thus, the post-symbiotic form should not be called “bacteroids”, as they no longer possess two of the key features of the symbiosis, nitrogenase and a living host partner. However, like bacteroids, the post-symbiotic form(s) of the bacteroid do not display any of the proteins or processes consistent with cellular growth and division, but they can be extracted from senescing nodules and grown on artificial medium [3,4,5,6,7,34,35].

Studies during the developmental time course of *B. diazoefficiens* bacteroids through symbiosis have followed several enzymes during the symbiosis, including nitrogenase, citric acid cycle enzymes, and the carbon storage compound poly-β-hydroxybutyrate [21,36]. These enzymes constitute the fixing of atmospheric dinitrogen, the energy metabolism for nitrogen fixation, and the storage of carbon metabolites in the determinate nodule system. Other studies have looked at the effects of mutations in hydrogenase systems on nitrogen fixation, leghemoglobin content, and nodule physiology up to 71 days after emergence [37]. Beyond these studies, there is no knowledge at present about the changes that the *B. diazoefficiens* bacteroids experiences during its re-differentiation to a free-living bacterium in the post-symbiotic state [3,4,5,6,7,36,37]. The enzymatic and proteomic analysis reported here and the transcriptomic analysis [33] provide insight into the physiological nature of the post-symbiotic form of *B. diazoefficiens*. The retention of metabolic and transcriptional [33] activity of the bacteria as the plant cells dies is the definition of hemibiotrophy [8,9]. A hemibiotroph is defined as an organism that is saprophytic or parasitic in living tissue while the plant is alive, and which upon plant death consumes the dead tissue [8,9]. Although a symbiont and not a parasite, *B. diazoefficiens* survives on plant-supplied metabolites during symbiosis and remains viable by consuming decaying plant compounds. *B. diazoefficiens* should be considered a highly specialized hemibiotroph, as it is restricted to limited plant hosts and a single, specialized plant organ, the nodule formed via symbiosis. The specificity of the infection process, and sequestration of the symbiont within the senescing nodule, has apparently limited the expression of the hemibiotrophic lifestyle of *B. diazoefficiens*, as it is not known to be a necrotroph on other plants.

The senescing nodule would be a metabolite-rich environment, with active proteases from the plant cells providing amino acids and peptides as metabolites [38]. A number of enzymatic and transport activities were identified among the constitutive and up-regulated proteins, suggesting the post-symbiotic form of *B. diazoefficiens* was accumulating and hydrolyzing peptides from the decaying plant nodule cells (Table 2 and Table 3). A previous study of 28-day-old, greenhouse-grown *B. diazoefficiens* bacteroids, at the period of maximal nitrogenase activity, indicated no defined fatty acid metabolism [12]. Fatty acid metabolism was markedly increased in the post-symbiotic period (Table 2). The symbiosome membrane amounts to approximately 30 times more membrane than that of the plasma membrane [39]. The turnover of membrane lipids derived from the senescing plant cell (both symbiosome and plasma membrane) could provide a rich source of energy for the post-symbiotic, hemibiotrophic-like *B. diazoefficiens*.

The bacteroids of winged bean appear to be protected from degradation via a 21 kDa nodulin that is homologous to a plant Kunitz trypsin inhibitor [40]. This raises the issue that not only is the senescing nodule a source of nutrients, but also a source of potentially harmful hydrolases from which the post-symbiotic bacteria need protection. The pyrroloquinoline quinone (PQQ)-dependent alcohol dehydrogenase (Table 3) further supports a role in bacterial protection, as it was previously found to be one of three PQQ-dependent dehydrogenases induced during osmotic stress [25]. The presence of CheY suggests that post-symbiotic bacteria are able to respond to the changing conditions within the senescing nodule (Table 2). The presence of enzymes in reactive oxygen metabolism suggests a protective mechanism against these species, generated during plant nodule senescence (Table 3).

The senescence of the plant nodule cells leads to the loss of functional symbiosome and plant plasma membranes and, thus, the selectivity of metabolites transported to bacteria is no longer restricted, as the bacterial periplasm must adapt to the diversity of metabolites produced from plant cell degradation. For example, during symbiosis, the bacteroids receive malate from the plant. Post-symbiotically, the source of malate and of dicarboxylates change. Curiously, the protocatechuate 3,4-dioxygenase and malate dehydrogenase activities display inverse patterns between 56 and 119 days in both periplasm and cytoplasmic fractions (Figure 6 and Figure 7). The two metabolic sources of dicarboxylates, represented by protocatechuate dioxygenase and malate dehydrogenase, are inversely regulated to maintain a constant source of dicarboxylates as the nodule environment changes.

Combined activity profiles of the enzymes, and transcripts [33] of the post-symbiotic bacteria each demonstrate unique patterns, suggesting that post-symbiotic bacteria are actively and purposely expressing metabolic responses to its changing environment. The presence of heat shock (blr0678), cold shock (bsl1386), and peptidyl prolyl cis-trans isomerase (bll5690) suggest the bacteria have the means for the remodeling of the bacteroid as it transitions to the post-symbiotic bacteria (Table 2 and Table 3). However, the 30S ribosomal proteins S1, S4, S7, and S18 and the 50S ribosomal proteins L14 showed a decrease beyond 91 days (Table 1). Franck et al. [33] showed the transcripts for the ribosomal proteins remain fairly constant up to 78 days, but some ribosomal proteins, particularly 30S ribosomal proteins S3, S21, S10, S17, and 50S ribosomal proteins L16 and L30 declined at 95 days after planting. This indicates a loss of overall translational activity despite the increase of select proteins at the last time point (data not shown).

Levels of poly-β-hydroxybutyrate (PHB), the major storage compound in determinate nodules, but not indeterminate nodules [41], were stable over the majority of the time course and then surprisingly increased nearly 3-fold between days 104–112 (Figure 2), without a corresponding change in β-hydroxybutyrate dehydrogenase activity (Figure 3). The reduction of translational activity combined with the increase in PHB suggest the bacteria may have reached a state of nutrient exhaustion and/or the build-up of waste products and will enter a quiescent state until the bacteria can be released from lignified nodule exterior and returned to the soil.

Three soybean proteins, histone H4, histone H3, glu/leu/phe/val dehydrogenase (Glyma02g38920.1, Glyma06g32880.1, Glyma16g04560.1), were present in the periplasm throughout the time course (Table 3). Previously, it was demonstrated that histone H2A and lipoxygenase were localized to the bacteroid surface [18], suggesting a role for these proteins. The presence of the soybean proteins in the periplasmic fractions of the post-symbiotic form of *B. diazoefficiens* suggests a role for these proteins and implies a continuation, albeit limited to a few specific intermolecular interactions, of the symbiosis among the two former symbionts beyond the period of nitrogen fixation.

In summary, the post-symbiotic form of *B. diazoefficiens* remains transcriptionally, translationally, and metabolically active late into senescence. During senescence, *B. diazoefficiens* transitions to a hemibiotrophic-like species that may still benefit from soybean-derived proteins and membranes.

## 4. Materials and Methods

### 4.1. Source of Nodules and Bacteroid Preparations

Soybean plants were obtained from the Bradford Research and Extension Center of the University of Missouri over a nine-week period (56–119 days after planting). *B. diazoefficiens* strains were residual and the seeds were not inoculated prior to planting. Soybean plants were harvested from the same, un-irrigated field between 8 and 9 a.m. Approximately 100 plants were harvested at each sampling. Intact roots and nodules attached to the tap root were placed in ice water and then harvested at 4 °C. Bacteroids were isolated as described previously [17] and enzyme activities were performed on the same day. Cytoplasmic and periplasmic fractions were prepared as described previously [42]. Briefly, 30 g of bacteroids from each biological replicate were aliquoted into 10 g amounts and resuspended in 10 mL of 25 mM citrate buffer, pH 4.0. After incubation at room temperature for 15 min, they were centrifuged at 12,000× *g* for 15 min and the pellet was gently brought up into isolation buffer (50 mM Tris-HCl, pH 7.5 with 2 mM EDTA and 20% (*w*/*v*) sucrose) using a #4 tapered-tip artist paint brush. The suspension was treated with ready-lyse lysozyme solution (25,000 U, Epicentre-Madison, WI, USA) and protease inhibitor cocktail (10 uL, Calbiochem, Rockland, MA, USA), mixed gently, and then incubated for 30 min at room temperature. The periplasm was obtained by centrifugation at 12,000× *g* for 10 min at 4 °C. The pelleted bacteroid spheroplasts were gently suspended in 15 mL of MEP buffer (5 mM MgCl_2_, 1 mM EDTA, 50 mM K-phosphate buffer, pH 7.0) to which 10 uL of protease inhibitor cocktail was added. The spheroplasts were then ruptured in the French press [17].

### 4.2. Enzymatic, Leghemoglobin, and Poly-β-hydroxybutyrate (PHB) Analysis

To ascertain the level of purity of the bacteroid periplasmic fraction, several enzymes known to be cytoplasmic were measured in both fractions as well as cyclic phosphodiesterase, a known periplasmic marker, to assay the amount of the periplasmic release, as previously outlined for rhizobia and bradyrhizobia bacteroids [43]. Also measured for activity was the possible periplasmic enzyme protocatechuate 3,4-dioxygnease. Procedures for measurement of enzymes were described previously [17], except protocatechuate 3,4-dioxygenase, which was measured by adding 50 μL of enzyme extract to 900 μL of 50 mM CHES, pH 9.3, and 50 μL of 40 mM protocatechuate and recording absorbance at 290 nm (molar absorptivity 3.8 mM^−1^ cm^−1^). Leghemoglobin concentration was measured using Drabkin’s reagent [44]. Poly-β-hydroxyburyrate (PHB) was measured as described by Karr et al. [45]. The large fluctuations of the data can be attributed to weather over particular sampling periods, but no effort was made to adjust the data accordingly.

### 4.3. Protein Isolation and Identification

The periplasmic and cytoplasmic fractions were each precipitated using equal volumes of phenol. The fractions were mixed at room temperature for one hour. Phases were then separated by centrifugation at 4000× *g* for 10 min at 4 °C. The phenol phase was collected and four volumes of 100% methanol containing 0.1 M ammonium acetate and 10 mM dithiothreitol added. Protein was precipitated overnight at −20 °C. Protein precipitate was collected by centrifugation at 4000× *g* for 10 min at 4 °C. The protein pellet was washed once with the methanol/ammonium acetate/dithiothreitol (DTT) solution. The protein pellet was then washed three times with 90% ethanol containing 10 mM DTT and then stored in 90% (*v*/*v*) ethanol/DTT at −80 °C.

Precipitated protein in 90% (*v*/*v*) ethanol/DTT was collected by centrifugation at 4000× *g* and 4 °C. Reconstitution buffer (30 mM Tris-HCl, 7 M urea, 2 M thiourea, 4% (*w*/*v*) CHAPS at pH 8.8) was added to the pellet, followed by gentle vortexing for one hour. A 20 µg portion of protein from each sample, quantified by the Bradford method, was removed and diluted to 1 µg/uL with reconstitution buffer. Bovine serum albumin was added as an internal standard to give a protein ratio of 1% (*w*/*w*).

Disulfide bonds were reduced with 10 mM DTT (100 mM stock in 50 mM ammonium bicarbonate), at 25 °C for 1 h and then alkylated with 40 mM iodoacetamide (200 mM stock in 50 mM ammonium bicarbonate), at 25 °C in the dark for 1 h, and finally quenched with additional DTT to 30 mM (100 mM stock in 50 mM ammonium bicarbonate) and incubated at 25 °C for 30 min. Urea was brought to 1 M by dilution with 50 mM ammonium bicarbonate. Trypsin (Sequencing Grade Modified-Promega, Madison, WI, USA) was reconstituted to 0.02 ug/uL and activated as per the manufacturer’s instructions in the provided resuspension buffer (1:200 *w*/*w*, trypsin:sample). Samples were incubated at 37 °C for 16 h. Digests were then lyophilized to dryness.

### 4.4. Mass Spectrometry Analysis

Lyophilized protein samples were reconstituted in 100 uL of 18 MΩ water with 0.1% (*v*/*v*) formic acid and 5.0% (*v*/*v*) acetonitrile by pipetting and mild vortexing. Samples were spun at 13,000 rpm at 4 °C for 10 min in a tabletop centrifuge to remove insoluble debris. Twenty uL portions from each sample were placed in polypropylene 96 v-well plates and covered with adhesive film. The plates were centrifuged to collect samples at the bottom of the well, and then placed in the precooled tray of the LC autosampler. Ten 10 µL injections were analyzed on a LTQ ProteomeX linear ion trap LC-MS/MS instrument (Thermo Fisher, San Jose, CA, USA). C_8_ captraps (Michrom Bioresources, Auburn, CA, USA) were used to concentrate and desalt peptides before final separation by C_18_ column chromatography (acetonitrile gradient of 0–90% solvent B (100% acetonitrile with 0.1% (*v*/*v*) formic acid), in solvent A (deionized 18 MΩ water with 0.1% (*v*/*v*) formic acid for a duration of 110 min). The peptide trap and C_18_ column were re-equilibrated with 100% solvent A for 25 min before applying the next sample.

LC separation was performed using fused silica nanospray needles (26 cm length, 360 μm outer diameter, 150 μm inner diameter; Polymicro Technologies, Phoenix, AZ, USA), packed with “Magic C18” (200 Å, 5 μm particles, Michrom Bioresources) in 100% methanol. Columns were equilibrated for 3–4 h at 200 nL/min (at the column tip) with a 60:40 mix of solvent B to solvent A prior to sample application. Samples analysis was performed in the data-dependent positive acquisition mode on the LC-MS/MS instrument, with a normal scan rate for precursor ion analysis with dynamic exclusion enabled (1 repeat count, 30 s repeat duration, 30 s exclusion, list size of 50). After each full scan (400–2000 *m*/*z*), a data-dependent triggered MS/MS scan was obtained for the three most intense parent ions. The nanospray column was maintained at ion sprays of 2.0 kV.

### 4.5. Database Searching and Spectral Analysis

Tandem mass spectra were obtained using BioWorks version 3.3. SEQUEST (ThermoFinnigan, San Jose, CA, USA; version 2.7) was set up to search a FASTA file containing translated coding regions for both *B. diazoefficiens* and *G. max*, with a concatenated random database with fragment ion mass tolerance of 1.0 Da and a parent ion tolerance of 2.0 Da. These protein sequence files, named “brady.p.aa.gz” “Gmax_109_peptide.fa.gz”, were provided at the websites (ftp://ftp.kazusa.or.jp/pub/RhizoBase/Bradyrhizobium/) and (ftp://ftp.jgi-psf.org/pub/JGI_data/phytozome/), respectively. The random concatenated database was generated using the tool “DecoyDBCreateor”, found at (http://p3db.org/p3db1.0/tools/DecoyDBCreator/). Iodoacetamide derivative of cysteine (+57) was specified as a fixed modification. RAW files were analyzed using Bioworks “SEQUEST batch-search”, with 10 matches reported without redundancy reporting. Data are available via ProteomeXchange with identifier: PXD011604 (https://www.ebi.ac.uk/pride/archive/). SEQUEST results were saved as .srf files.

### 4.6. Peptide Match Filtering

In order to filter the SEQUEST matches for nonrandom hits, the files were converted to “.SQT” file formats. Filtering of the “.SQT” files was performed using SEPro (Warrendale, PA, USA) [22] with the following settings: Spectral FDR: 5%, Peptide FDR: 3%, and final filtering of protein hits at: 1%. All filtered data were saved as SEPro file outputs (.spr).

### 4.7. Protein Expression Trends

Spectral count data associated with the protein IDs provided by SEPro were used for trend analysis via the proteomic analysis software PatternLab [46]. As per the software workflows, PatternLab input files were created using the Regrouper software (Pittsburgh, PA, USA) (SparseMatrix and index files). Folders for each time point in the time course were created, and the selected SEPro files for each timepoint were placed into the folders. Regrouper was pointed to these folders, and the SparseMatrix and index files were created. These files were provided to PatternLab in the TrendQuest module. Trends were created using an assigned minimum average signal of 2 per 6 replicates (3 biological, 2 technical), with minimum data points of 2 and minimum items per cluster of 3 and a health of 0.800.

## Figures and Tables

**Table 1:** Proteins identified in cytoplasmic and periplasmic fractions of *B. diazoefficiens* isolated from soybean nodules that declined from symbiosis through senescence.

Function	Rhizobase	Accession Number	Location
67 kDa Myosin-cross-reactive streptococcal antigen homolog	bll0025	NP_766665	Cytoplasm
Dihydrolipoamide dehydrogenase	bll0449	NP_767089	Cytoplasm
Succinyl-CoA synthetase beta chain	bll0455	NP_767095	Cytoplasm
Malate dehydrogenase	bll0456	NP_767096	Cytoplasm
3-isopropylmalate dehydrogenase	bll0504	NP_767143	Cytoplasm
Protein-export protein	bll0641	NP_767281	Cytoplasm
ABC transporter glycerol-3-phosphate-binding protein	bll0733	NP_767373	Cytoplasm
Polyribonucleotide nucleotidyltransferase	bll0779	NP_767419	Cytoplasm
ABC transporter substrate-binding protein	bll0887	NP_767527	Cytoplasm
Fructose bisphosphate aldolase	bll1520	NP_768160	Cytoplasm
Hypothetical protein	bll1875	NP_768515	Cytoplasm
GroEL3 chaperonin	bll2059	NP_768699	Both
GroES3 chaperonin	bll2060	NP_768700	Cytoplasm
phenolhydroxylase homolog	bll2063	NP_768703	Both
MoxR family protein	bll2465	NP_769105	Cytoplasm
Cystathionine beta lyase	bll4445	NP_771085	Cytoplasm
Hypothetical protein	bll4781	NP_771421	Cytoplasm
Pyruvate dehydrogenase beta subunit	bll4782	NP_771422	Cytoplasm
Enolase	bll4794	NP_771434	Cytoplasm
Translation elongation factor Ts	bll4860	NP_771500	Periplasm
Prolyl-tRNA synthetase	bll4877	NP_771517	Cytoplasm
ATP-dependent protease LA	bll4942	NP_771582	Cytoplasm
Trigger factor	bll4945	NP_771585	Cytoplasm
50S ribosomal protein L14	bll5390	NP_772030	Cytoplasm
Elongation factor TU	bll5402	NP_772042	Cytoplasm
Translation elongation factor G	bll5403	NP_772043	Cytoplasm
30S ribosomal protein S7	bll5404	NP_772044	Cytoplasm
DNA-directed RNA polymerase beta chain	bll5409	NP_772049	Cytoplasm
DNA-directed RNA polymerase beta chain	bll5410	NP_772050	Cytoplasm
Carbon monoxide dehydrogenase large chain	bll5914	NP_772554	Cytoplasm
Putative hydrolase serine protease transmembrane protein	bll6508	NP_773148	Cytoplasm
3-oxoadipate CoA-transferase subunit A	bll7093	NP_773733	Cytoplasm
DNA-binding stress response protein, Dps family	bll7374	NP_774014	Cytoplasm
Phosphoenolpyruvate carboxykinase	bll8141	NP_774781	Cytoplasm
Unknown protein	bll8309	NP_774949	Periplasm
Hypothetical protein	blr0028	NP_766668	Cytoplasm
3-isopropylmalate dehydratase large subunit	blr0488	NP_767128	Cytoplasm
Succinate dehydrogenase flavoprotein subunit	blr0514	NP_767154	Cytoplasm
30S ribosomal protein S1	blr0740	NP_767380	Cytoplasm
Catalase	blr0778	NP_767418.	Cytoplasm
Succinate-semialdehyde dehydrogenase	blr0807	NP_767447	Cytoplasm
Putative aminotransferase protein	blr1686	NP_768326	Both
Nitrogenase molybdenum-iron protein alpha chain (NifD)	blr1743	NP_768383	Both
Nitrogenase molybdenum-iron protein beta chain (NifK)	blr1744	NP_768384	Both
Flavoprotein (FixC)	blr1774	NP_768414	Both
Pantoate--beta-alanine ligase	blr1852	NP_768492	Cytoplasm
5-methyltetrahydropteroyltriglutamate-homocysteine S-methyltransferase	blr2068	NP_768708	Cytoplasm
Unknown protein	blr2372	NP_769012	Cytoplasm
Glutaryl-CoA dehydrogenase	blr2616	NP_769256	Cytoplasm
Aldehyde dehydrogenase	blr2816	NP_769456	Cytoplasm
Nucleoside diphosphate kinase	blr4119	NP_770759	Cytoplasm
Aminotransferase	blr4134	NP_770774	Cytoplasm
NAD-dependent malic enzyme	blr4145	NP_770785	Cytoplasm
Citrate synthase	blr4839	NP_771479	Both
Glutamine synthetase I	blr4949	NP_771589	Cytoplasm
Anti-oxidant protein	blr5308	NP_771948.	Cytoplasm
60 KDA chaperonin	blr5626	NP_772266	Both
Adenylosuccinate lyase	blr5690	NP_772330	Cytoplasm
30S ribosomal protein S4	blr5706	NP_772346	Cytoplasm
Hypothetical protein	blr6718	NP_773358	Cytoplasm
Hypothetical protein	blr7070	NP_773710	Cytoplasm
Hypothetical zinc protease	blr7485	NP_774125	Cytoplasm
Glutamate synthase large subunit	blr7743	NP_774383	Cytoplasm
ABC transporter substrate-binding protein	blr8117	NP_774757	Cytoplasm
Two-component response regulator	blr8144	NP_774784	Cytoplasm
30S ribosomal protein S18	bsl4078	NP_770718	Cytoplasm

**Table 2:** Proteins identified in cytoplasmic and periplasmic fractions of *B. diazoefficiens* isolated from soybean nodules that increased from symbiosis through senescence.

Function	Rhizobase	Accession	Location
Hypothetical protein	bll2012	NP_768652	Cytoplasm
Unknown protein	bll4278	NP_770918	Cytoplasm
Peptidyl prolyl cis-trans isomerase	bll4690	NP_771330	Cytoplasm
Hypothetical protein	bll5155	NP_771795	Cytoplasm
Hypothetical protein	bll7395	NP_774035	Cytoplasm
CheY, two-component response regulator	bll7795	NP_774435.	Periplasm
Probable fatty oxidation complex, alpha subunit	bll7821	NP_774461	Periplasm
Acetyl-CoA carboxylase carboxyl transferase α-subunit	blr0191	NP_766831	Cytoplasm
Carboxy-terminal protease	blr0434	NP_767074	Periplasm
Acyl-CoA thiolase	blr1159	NP_767799	Periplasm
Enoyl-CoA hydratase	blr1160	NP_767800	Periplasm
Unknown protein	blr1830	NP_768470	Cytoplasm
Hypothetical protein	blr3804	NP_770444	Cytoplasm
ABC transporter substrate-binding protein	blr4039	NP_770679	Periplasm
Hypothetical protein	blr5502	NP_772142	Cytoplasm
Cold shock protein	bsl3986	NP_770626	Cytoplasm

**Table 3:** Proteins identified in cytoplasmic and periplasmic fractions of *B. diazoefficiens* isolated from soybean nodules that remained unchanged from symbiosis through senescence.

Function	Rhizobase	Accession Number	Fraction
N-utilization substance protein A	bll0785	NP_767425	Periplasm
Transketolase	bll1524	NP_768164	Pperiplasm
Alkyl hydroperoxide reductase	bll1777	NP_768417	Cytoplasm
Carbonic anhydrase	bll2065	NP_768705	Cytoplasm
Nodulate formation efficiency C protein	bll2067	NP_768707	Periplasm
Probable amino acid binding protein	bll2909	NP_769549	Both
Glycine hydroxymethyltransferase	bll5033	NP_771673	Periplasm
ABC transporter substrate-binding protein	bll5596	NP_772236	Cytoplasm
RecA protein	bll5755	NP_772395	Periplasm
Hypothetical protein	bll6069	NP_772709	Periplasm
PQQ-dependent alcohol dehydrogenase	bll6220	NP_772860	Cytoplasm
Peptidyl-dipeptidase	bll7756	NP_774396	Periplasm
Superoxide dismutase	bll7774	NP_774414	Cytoplasm
ABC transporter substrate-binding protein (Putative oligopeptide binding protein)	bll7921	NP_774561	Both
Hypothetical protein	bll8229	NP_774869	Periplasm
Heat shock protein 70	blr0678	NP_767319	Periplasm
Beta-ketoadipyl CoA thiolase	blr0925	NP_767565	Periplasm
Peptidoglycan acetylation protein	blr0936	NP_767576	Periplasm
ATP-dependent protease, ATP-binding subunit	blr1404	NP_768044	Periplasm
Dehydrogenase	blr2146	NP_768786	Periplasm
Methylmalonate-semialdehyde dehydrogenase	blr3954	NP_770594	Periplasm
Probable transaldolase	blr6758	NP_773398	Periplasm
Histone H4	-	Glyma02g38920	Periplasm
Histone H3	-	Glyma06g32880	Periplasm
Glu/leu/phe/val dehydrogenase	-	Glyma16g04560	Periplasm
